# Dihydrofolate Reductase Is a Valid Target for Antifungal Development in the Human Pathogen *Candida albicans*

**DOI:** 10.1128/mSphere.00374-20

**Published:** 2020-06-24

**Authors:** Christian DeJarnette, Arturo Luna-Tapia, Leanna R. Estredge, Glen E. Palmer

**Affiliations:** a Department of Molecular Immunology and Biochemistry, College of Graduate Health Sciences, University of Tennessee Health Science Center, Memphis, Tennessee, USA; b National Program in Biotechnology, Ministry of Science, Technology and Innovation, Bogota, Colombia; c Department of Clinical Pharmacy and Translational Science, College of Pharmacy, University of Tennessee Health Science Center, Memphis, Tennessee, USA; University of Georgia

**Keywords:** *Candida albicans*, antifungal agents, dihydrofolate reductase

## Abstract

While the folate biosynthetic pathway has provided a rich source of antibacterial, antiprotozoal, and anticancer therapies, it has not yet been exploited to develop uniquely antifungal agents. Although there have been attempts to develop fungal-specific inhibitors of dihydrofolate reductase (DHFR), the protein itself has not been unequivocally validated as essential for fungal growth or virulence. The purpose of this study was to establish dihydrofolate reductase as a valid antifungal target. Using a strain with doxycycline-repressible transcription of *DFR1* (*P_TETO_-DFR1* strain), we were able to demonstrate that Dfr1p is essential for growth *in vitro*. Furthermore, nutritional supplements of most forms of folate are not sufficient to restore growth when Dfr1p expression is suppressed or when its activity is directly inhibited by methotrexate, indicating that Candida albicans has a limited capacity to acquire or utilize exogenous sources of folate. Finally, the *P_TETO_-DFR1* strain was rendered avirulent in a mouse model of disseminated candidiasis upon doxycycline treatment. Collectively, these results confirm the validity of targeting dihydrofolate reductase and, by inference, other enzymes in the folate biosynthetic pathway as a strategy to devise new and efficacious therapies to combat life-threatening invasive fungal infections.

**IMPORTANCE** The folate biosynthetic pathway is a promising and understudied source for novel antifungals. Even dihydrofolate reductase (DHFR), a well-characterized and historically important drug target, has not been conclusively validated as an antifungal target. Here, we demonstrate that repression of DHFR inhibits growth of Candida albicans, a major human fungal pathogen. Methotrexate, an antifolate, also inhibits growth but through pH-dependent activity. In addition, we show that C. albicans has a limited ability to take up or utilize exogenous folates as only the addition of high concentrations of folinic acid restored growth in the presence of methotrexate. Finally, we show that repression of DHFR in a mouse model of infection was sufficient to eliminate host mortality. Our work conclusively establishes DHFR as a valid antifungal target in C. albicans.

## Abstract (precis)

The folate biosynthetic pathway is a promising and understudied source for novel antifungals. Even dihydrofolate reductase (DHFR), a well-characterized and historically important drug target, has not been conclusively validated as an antifungal target. Here, we demonstrate that repression of DHFR inhibits growth of Candida albicans, a major human fungal pathogen. Methotrexate, an antifolate, also inhibits growth but through pH-dependent activity. In addition, we show that C. albicans has a limited ability to take up or utilize exogenous folates as only the addition of high concentrations of folinic acid restored growth in the presence of methotrexate. Finally, we show that repression of DHFR in a mouse model of infection was sufficient to eliminate host mortality. Our work conclusively establishes DHFR as a valid antifungal target in C. albicans.

## INTRODUCTION

The CDC estimates that the direct health care costs of treating fungal infections to be over $7 billion each year in the United States alone (1). Along with this high financial burden, over 1 million deaths are attributed to invasive fungal infections (IFIs) annually (2), despite appropriate use of the available antifungal drugs. The most widely used antifungal drug class, the azoles, inhibit ergosterol biosynthesis, but the incidence of resistance is on the rise and starting to compromise their clinical utility (3). The polyene amphotericin B disrupts fungal membranes through direct interaction with ergosterol (4). However, despite broad-spectrum antifungal activity, this drug is associated with severe patient toxicity that limits its use. The most recently approved antifungal drugs are the echinocandins, which target cell wall biosynthesis. Drugs in this class are principally active against *Candida* species and therefore have a narrow spectrum of activity and are available only in intravenous formulations, further limiting their clinical utility (5). A larger concern is the modest efficacy of all three classes of antifungal drugs as this is likely a major determinant of the excessively high mortality rates in patients with IFIs, as well as the high rates of recurrent mucosal infections. For example, approximately one-third of patients with disseminated *Candida* infections are nonresponsive to treatment with fluconazole (6–10), voriconazole (11), or the recently approved isavuconazole (12), even among those infected by isolates deemed susceptible according to current clinical breakpoints. Favorable response rates of just 52% to 73% have been reported for patients with disseminated candidiasis who are treated with an echinocandin (12–16) and of 62% for those treated with amphotericin B (14, 16). Of grave concern is the recent isolation of Candida auris from patients across the globe that is resistant to all three classes of antifungal drugs (17). As such, there is an urgent need for new antifungal drugs with improved therapeutic efficacy, patient safety, and spectrum of activity.

In its reduced form, tetrahydrofolate (THF) is an essential coenzyme for a number of cellular enzymes, serving as a carrier for the transfer of one-carbon (1C) units as well as their interconversion between various oxidation states (18). THF is required for the synthesis of dTMP, purines, and methionine, as well as a multitude of other important metabolites. Mammals are unable to synthesize folate (FOL) as they lack several of the necessary enzymes and must acquire it through dietary intake. A specialized transport system is required for cellular uptake of folic acid, which is then converted into its active form, THF, by dihydrofolate reductase (DHFR) (19). In contrast, the majority of prokaryotes and microbial eukaryotes lack the transport systems found in mammals and must therefore synthesize folic acid *de novo*.

The folate biosynthetic pathway has been targeted with enormous success in the development of antineoplastic, antibacterial, and antiprotozoal drugs. Anticancer drugs include folate analogs such as methotrexate (MTX), a potent inhibitor of DHFR (20, 21). Other notable antifolates include trimethoprim, which inhibits bacterial DHFR, and a collection of sulfa drugs, including sulfamethoxazole, that inhibit dihydropteroate synthase (DHPS) from some bacterial as well as protozoan parasites (22). Antiprotozoal drugs that target this pathway have been especially important for the treatment of malaria and include the DHFR inhibitors pyrimethamine, proguanil (PRO), and chlorproguanil (23–25), as well as the DHPS inhibitor dapsone (26). Curiously, a combination of trimethoprim and sulfamethoxazole can also provide an effective treatment for *Pneumocystis* pneumonia caused by the atypical fungus Pneumocystis jirovecii (27). Although these drugs were not specifically developed to target the folate biosynthetic enzymes of *Pneumocystis*, they provide clinical evidence that targeted inhibition of the folate (FOL) pathway can provide an effective intervention strategy to treat invasive mycoses. However, the conventional antifolate drugs developed for bacterial or protozoan DHPS or DHFR, or even human DHFR, have little or no antifungal activity upon the major human fungal pathogens (28–30), either because of divergence of the fungal enzyme structures or permeability issues that prevent antifolates from entering fungal cells (28). Furthermore, until now, no published study has unequivocally determined if the core components of the FOL pathway are required by infectious fungi to cause disease within a mammalian host and are therefore valid and potentially efficacious targets for antifungal development. The objective of this study was to determine if DHFR is essential for Candida albicans, one of the most prevalent human fungal pathogens, to survive and cause disease within its mammalian host.

## RESULTS

### DHFR is essential for Candida albicans growth *in vitro*.

**FIG 1:**
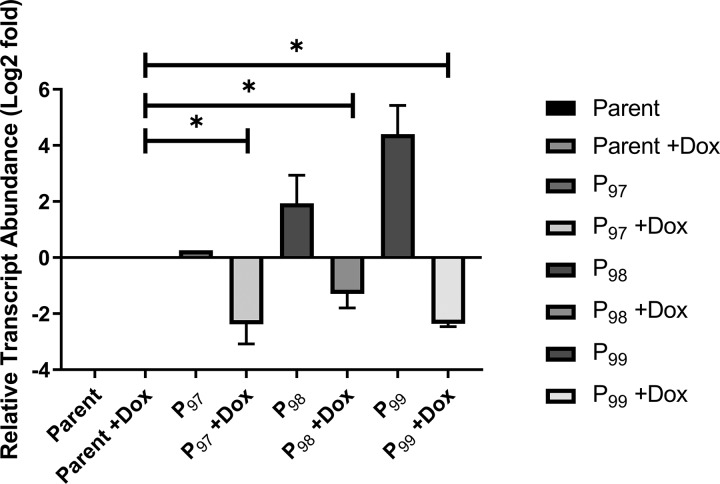
Analysis of *DFR1* transcript expression in Candida albicans *P_TETO_-DFR1* strains. C. albicans strains of the indicated genotypes were grown to exponential phase in YPD medium in the presence or absence of 5 μg/ml doxycycline (DOX). Total RNA was extracted, and *DFR1* transcript abundance determined by qRT-PCR. *DFR1* transcript abundance was normalized to that of the *ACT1* transcript and then expressed relative to the *DFR1* transcript abundance in the wild-type control (SC5314) in the absence of doxycycline. These data are the average of three biological and technical replicates. *, *P* < 0.0001 (for results compared to those with the parent strain under the same condition using an unpaired Student's *t* test). P_97_, *P_TET97_-DFR1* strain; P_98_, *P_TET98_-DFR1* strain; P_99_, *P_TET99_-DFR1* strain.

To investigate the importance of DHFR in C. albicans, we generated strains with doxycycline-repressible transcription of *DFR1*, which encodes DHFR (31). One allele of *DFR1* was deleted from a strain expressing the tetracycline-responsive transactivator protein and replaced with the *ARG4* selection marker (32). The promoter of the second allele was then replaced with one of three tetracycline-repressible promoters, *P_TETO97_*, *P_TETO98_*, or *P_TETO99_*, which support different basal levels of transcription (33). *DFR1* transcript abundance in strains of each genotype was then compared to that of a wild-type control (*DFR1*/*DFR1*) by quantitative reverse transcription-PCR (qRT-PCR) (Fig. 1). In the absence of doxycycline, the *P_TET97_-DFR1* strain produced levels of the *DFR1* transcript similar to those of the wild-type control, while the *P_TET98_-DFR1* and *P_TET99_-DFR1* strains overproduced the *DFR1* transcript by ∼4- and ∼20-fold, respectively. However, in the presence of 5 μg/ml doxycycline, the *DFR1* transcript abundance was dramatically reduced in strains of all three genotypes to below that of the wild-type control (Fig. 1). We noted that the *P_TET97_-DFR1* strain grew slowly compared to growth of the wild type, even in the absence of doxycycline, suggesting that Dfr1p production is insufficient, while both the *P_TET98_-DFR1* and *P_TET99_-DFR1* strains grew at rates comparable to the wild-type rate (see Fig. S1 in the supplemental material). We, therefore, selected the *P_TET98_-DFR1* strain for further study.

10.1128/mSphere.00374-20.1 FIG S1 The *dfr1Δ*/*P_TETO97_*-*DFR1* strain has a marginal growth defect. The indicated strains were subcultured into YPD broth at approximately 5 × 10^5^ cells/ml and dispensed into the wells of a 96-well plate. The plate was incubated at 30°C for 48 h, and growth was monitored at 30-min intervals as the OD_600_. The values presented here are the average of 24 replicates in a single experiment. Download FIG S1, TIF file, 0.6 MB. Copyright © 2020 DeJarnette et al. 2020 DeJarnette et al. This content is distributed under the terms of the Creative Commons Attribution 4.0 International license.

**FIG 2:**
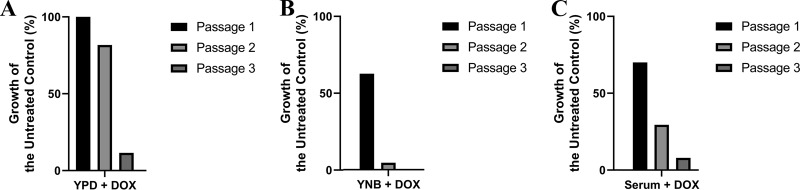
Dfr1p is essential for Candida albicans growth *in vitro*. A *dfr1Δ*/*P_TETO98_-DFR1* strain was sequentially passaged in YPD broth (A), YNB broth (B), or fetal bovine serum (C), each supplemented with 5 μg/ml doxycycline (DOX). Following inoculation at approximately 5 × 10^5^ cells/ml, each culture was incubated at 30°C for 24 h, and growth was measured after 24 h as the OD_600_ value. Relative growth was then expressed as a percentage of the untreated control, i.e., without doxycycline. The values presented here are representative of two independent experiments.

Next, we determined if *DFR1* expression is required for C. albicans growth *in vitro*. When inoculated into rich yeast extract-peptone-dextrose (YPD) medium supplemented with 5 μg/ml doxycycline, the *P_TET98_-DFR1* strain grew to the same cell density as in the absence of doxycycline, and to the same density as the wild-type control (Fig. 2A). However, when the strain was sequentially passaged in the presence of doxycycline, growth was dramatically reduced by the third passage. This suggests that *DFR1* is essential for C. albicans growth, even in nutritionally rich growth medium containing high concentrations of folate as well as metabolites that depend upon folate for their biosynthesis. The prolonged lag between doxycycline exposure and growth suppression could indicate that cellular stores of THF permit continued growth in the absence of Dfr1p until THF is depleted or that the protein has a long half-life. To distinguish between these possibilities, we attempted to directly inhibit Dfr1p using methotrexate. Previous reports have indicated that MTX lacks antifungal activity against C. albicans (28–30), despite potent inhibition of purified fungal DHFR in biochemical assays (34). However, we recently reported that MTX has relatively potent and on-target activity upon whole C. albicans cells in unbuffered yeast nitrogen base (YNB) medium (35). We therefore examined how MTX affected the growth of a wild-type C. albicans strain in YNB medium in dose-response assays. Significant growth inhibition was observed in this medium, with a MIC_50_ of approximately 0.78 μM (data not shown), without the need for passaging. In addition, passaging of the *P_TET98_-DFR1* strain with doxycycline in YNB medium resulted in detectable growth inhibition without the need for passaging (Fig. 2B). These results suggest that the continued growth of the *P_TET98_-DFR1* strain upon exposure to doxycycline is likely not due to the utilization of an intracellular store of THF. Instead, these results are consistent with the Dfr1p enzyme having a long half-life, resulting in a long lag before doxycycline-mediated suppression arrests fungal growth.

### Candida albicans has a limited capacity to utilize exogenous sources of folate.

**FIG 3:**
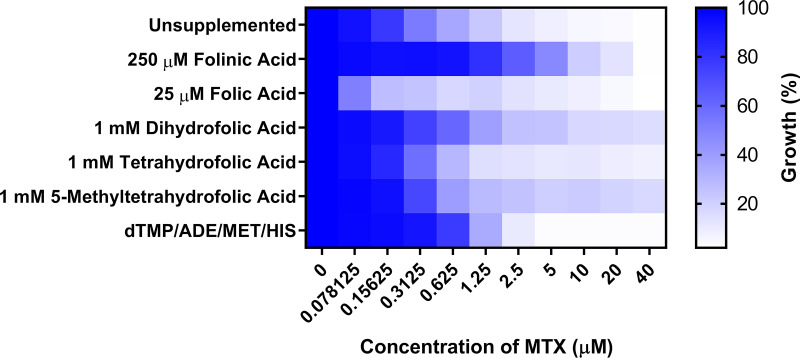
Excess folinic acid partially restores the growth of Candida albicans in the presence of methotrexate. A wild-type C. albicans strain (SC5314) was seeded at 1 × 10^4^ cells/ml into YNB medium without folate or PABA with the indicated supplements and increasing concentrations of MTX. After 48 h of incubation at 30°C, growth was quantified as the OD_600_ value and expressed as a percentage of the value for the DMSO-treated control. The values presented here are the average of two replicates and are representative of two independent experiments.

**FIG 4:**
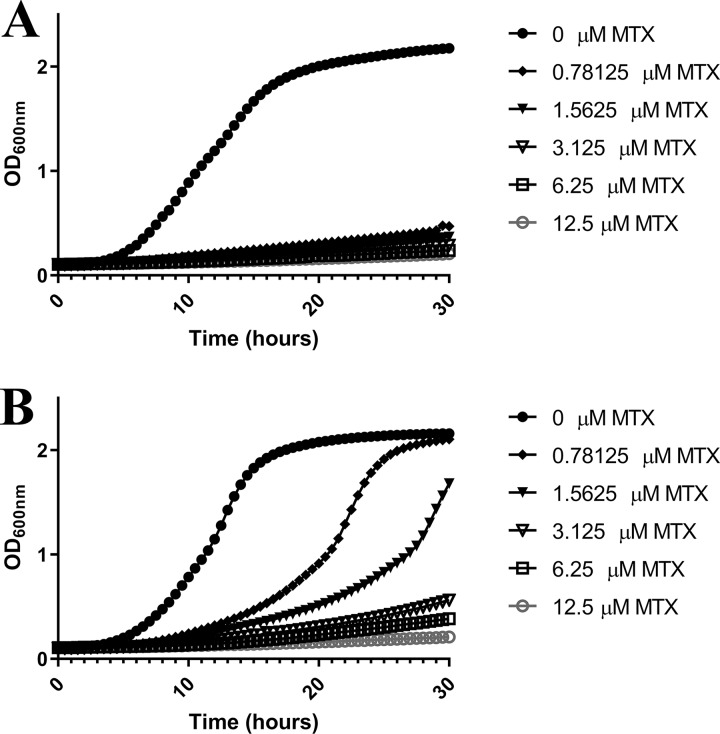
Folinic acid restores growth of Candida albicans in the presence of methotrexate. C. albicans strain SC5314 was subcultured into YNB medium (A) or YNB medium supplemented with 500 μM folinic acid (B) at ∼5 × 10^5^ cells/ml in the presence of increasing concentrations of MTX. These cultures were established in 96-well plates to facilitate the monitoring of growth at 30-min intervals as the OD_600_ value during a 48-h incubation period at 30°C. The values presented here are representative of two independent experiments.

Folate biosynthesis-deficient mutants of the yeast Saccharomyces cerevisiae are unable to grow in standard lab medium (36, 37). However, growth can be restored by supplementing the medium with extremely high concentrations (≥250 μM) of folinic acid (5-formyltetrahydrofolate) (38). To determine if exogenous sources of folate are sufficient to support C. albicans growth in the absence of DHFR activity, we seeded strain SC5314 into unbuffered YNB medium supplemented with either folic acid, dihydrofolate (DHF), THF, or 5-methyltetrahydrofolate (5MTHF) and compared growth in the presence of different concentrations of MTX. Notably, standard YNB medium contains both folic acid (4.5 nM) and *para*-aminobenzoic acid (PABA), a precursor required for folate biosynthesis. Therefore, MTX sensitivity was also tested in folate- and PABA-free YNB medium. The MIC_50_ of MTX in standard YNB medium (0.78 μM) was similar to that in the folate- and PABA-free medium, indicating that the folate present in standard YNB medium is not sufficient to affect sensitivity. Additional supplements of folic acid were completely unable to rescue C. albicans growth at any soluble concentration (up to 25 μM), and, in fact, the MIC_50_ of MTX dropped ∼10-fold to 0.078 μM (Fig. 3). Supplementing the medium with either 1 μM DHF, THF, or 5MTHF (Fig. S4) (well above blood serum concentrations) (39–41) had no effect on the MTX-mediated growth inhibition of C. albicans. However, the addition of 250 μM folinic acid to YNB was able to partially restore growth in the presence of MTX and elevated the MIC_50_ to ∼5 μM (Fig. 3). Nevertheless, even in the presence of this excess of folinic acid, MTX inhibited C. albicans growth in a dose-dependent manner (Fig. 4), indicating that the need for Dfr1p activity is not completely bypassed. Accordingly, we conclude that C. albicans is unable to take up and utilize sufficient quantities of exogenous folates to sustain growth in standard laboratory medium.

A combination of dTMP, adenine, histidine, and methionine, each of which requires folate for its biosynthesis, is sufficient to partially restore S. cerevisiae growth following treatment with antifolates (36). However, these supplements were not sufficient to restore C. albicans growth in the presence of MTX (Fig. 3). This suggests that either the provision of exogenous sources of folate-dependent metabolites is not adequate to bypass the need for *de novo* folate biosynthesis in C. albicans or that the nutritional requirements of this species in the absence of folate are more complex than those of S. cerevisiae. Finally, we examined if C. albicans was able to scavenge sufficient folates or folate-dependent metabolites from blood serum to negate the need for *de novo* biosynthesis. The *P_TET98_-DFR1* strain was passaged for 3 days in 100% fetal bovine serum (FBS) with or without doxycycline. In the first passage, doxycycline inhibited growth by ∼60%, with increasing growth inhibition seen in subsequent subcultures (Fig. 2C). Thus, C. albicans is unable to acquire sufficient nutrients from blood serum to bypass the need for *de novo* folate biosynthesis.

### *DFR1* is essential for Candida albicans virulence in a mouse model of disseminated infection.

**FIG 5:**
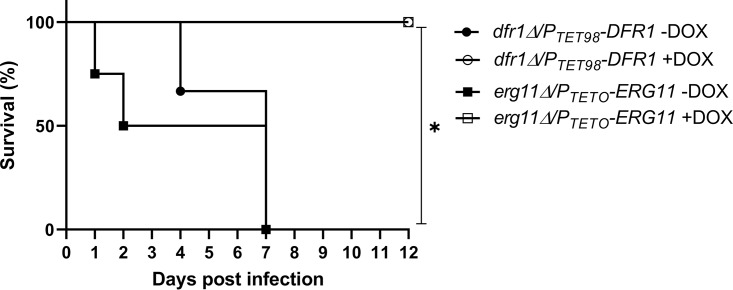
Dfr1p is essential for Candida albicans virulence in a mouse model of disseminated candidiasis. Groups of BALB/c mice (*n* = 6) were inoculated with ∼5 × 10^5^ CFU of either the *dfr1Δ*/*P_TET98_-DFR1* strain or the *erg11Δ*/*P_TETO_-ERG11* strain via the lateral tail vein injections. The Erg11p strain was included to compare the antifungal efficacy of targeting Dfr1p with a previously validated target. The mice were monitored three times daily for 12 days, and those showing signs of distress were humanely euthanized. Mice were treated with 2 mg/g doxycycline (DOX) or mock treated with vehicle alone provided in a gel food formulation from 72 h prior to infection and throughout the duration of the experiment. The survival of each group was compared using a log rank test (*, *P* < 0.0003).

Next, we determined if *DFR1* expression is essential for C. albicans virulence in a mammalian host using a mouse model of disseminated candidiasis. Female BALB/c mice were split into two treatment groups: group 1 was treated with doxycycline in a gel food formulation from 72 h before infection and for the duration of the experiment; group 2 was mock treated using unsupplemented gel food. The mice were then infected with ∼5 × 10^5^ yeast cells of the *P_TET98_-DFR1* strain via the lateral tail vein, and their health was monitored for 12 days. Animals exhibiting significant signs of distress were euthanized, and survival rates were compared. All mice in the mock-treated group succumbed to the infection by day 7 (Fig. 5A), confirming the virulence of the *P_TET98_-DFR1* strain under nonrepressing conditions. In stark contrast, all doxycycline-treated mice survived for the duration of the experiment. On day 12, surviving animals were euthanized, kidneys were extracted and homogenized, and levels of fungal colonization were determined as CFU counts. This revealed that 5 of the 8 surviving mice had undetectable levels of fungal colonization, essentially clearing the infection, with the remaining animals having extremely low CFU levels (data not shown). To determine if suppression of Dfr1p activity is sufficient to resolve an established infection, a second experiment was performed in which mice were infected with the *P_TET98_-DFR1* strain, and doxycycline treatment was initiated 24-h postinfection. Again, all mice survived the duration of the experiment (to day 12 postinfection), with initial symptoms resolving within 6 days of doxycycline treatment. Half of these mice had undetectable levels of fungal colonization in their kidneys at the end of the experiment (data not shown). These data confirm that Dfr1p is essential for C. albicans to survive within and cause disseminated disease in a mammalian host. Furthermore, C. albicans is unable to scavenge sufficient sources of folate to survive within mammalian tissue, and *de novo* biosynthesis is required. Indeed, the antifungal efficacy achieved upon repressing Dfr1p expression appears to be similar to that attained upon suppression of Erg11p expression. Accordingly, DHFR and likely other FOL pathway enzymes can provide potentially efficacious targets for antifungal development.

### The pH-dependent antifungal activity of methotrexate is likely due to differential uptake.

**FIG 6:**
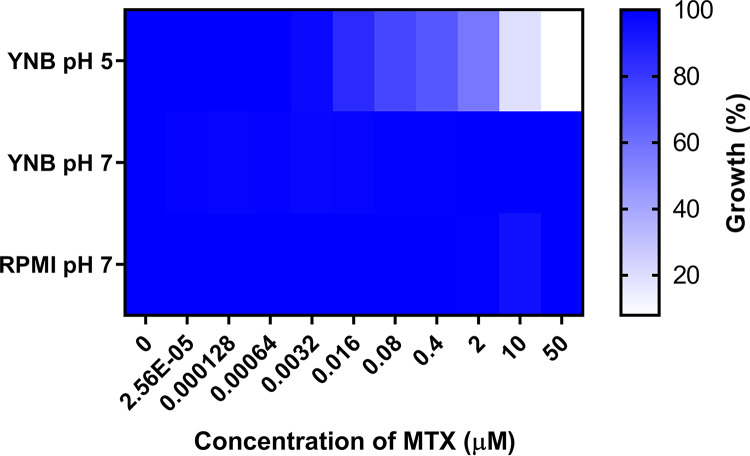
Methotrexate has pH-dependent antifungal activity on Candida albicans. Approximately 1 × 10^4^ cells/ml of a wild-type C. albicans strain was seeded into a 96-well plate with increasing concentrations of MTX in YNB pH 5, YNB pH 7, and RPMI pH 7 media. After 48 h, the OD_600_ was measured and expressed as a percentage of the value for the DMSO-treated control. The results presented here are the averages of two replicates and are representative of two independent experiments.

**FIG 7:**
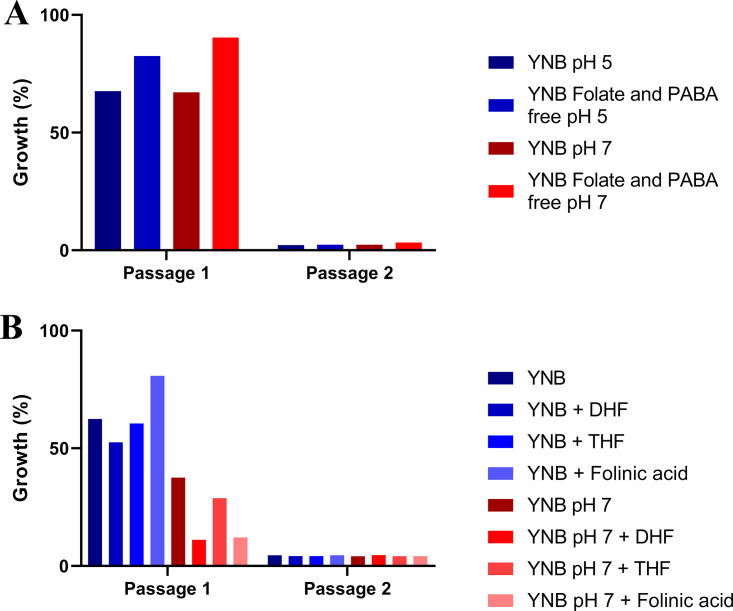
Dfr1p is essential for Candida albicans growth in both acidic and neutral conditions. The *dfr1Δ*/*P_TETO98_-DFR1* strain was passaged in YNB medium at pH 5 or 7 as well as the folate- and PABA-free YNB medium at the same pH (A) or in YNB medium at pH 5 or 7 supplemented with different forms of folate (B) with approximately 5 × 10^5^ cells/ml from the previous passage in the presence of 5 μg/ml doxycycline. The culture was incubated at 30°C in a rotating incubator, and after 24 h the OD_600_ was measured. The growth was calculated as a percentage of that of the untreated control, i.e., without doxycycline. The values presented here are representative of two independent experiments.

While MTX has been shown to inhibit fungal DHFR in cell-free biochemical assays using purified enzyme, it has also been reported to lack antifungal activity (28–30). Given that these previous studies used RPMI 1640 pH 7 medium, we sought to account for the antifungal activity we observed in unbuffered YNB medium. We first confirmed that MTX lacked antifungal activity in RPMI 1640 pH 7 medium (MIC_50_ > 50 μM) (Fig. 6). Next, to determine if the antifungal activity of MTX is pH dependent, dose-response experiments were conducted with strain SC5314 in YNB medium buffered at pH 5 or pH 7. MTX inhibited C. albicans growth in YNB pH 5 medium with a MIC_50_ of ∼0.78 μM (Fig. 6) but had no effect in YNB pH 7 medium (MIC_50_ >50 μM), indicating pH-dependent antifungal activity. Similar results were obtained in YNB medium lacking folate and PABA, buffered to either pH 5 or pH 7 (Fig. S2). In contrast, the *P_TET98_-DFR1* strain was unable to grow in regular YNB medium, in folate- and PABA-free YNB medium at either pH in the presence of doxycycline (Fig. 7A), or in YNB medium supplemented with different folates (Fig. 7B), indicating that Dfr1p expression and, by inference, *de novo* folate biosynthesis are essential at either pH. These data are consistent with the previous findings of Navarro-Martinez and colleagues that demonstrated that MTX does not accumulate within C. albicans yeast cells at neutral pH (28) and may therefore account for the pH-dependent antifungal activity.

10.1128/mSphere.00374-20.2 FIG S2 The presence of exogenous folate does not alter Candida albicans sensitivity to methotrexate. C. albicans strain SC5314 was seeded into YNB medium with or without folate buffered at pH 5 or pH 7 to approximately 1 × 10^4^ cells/ml with increasing concentrations of MTX. After 48 h at 30°C, growth was quantified as the OD_600_ and expressed as a percentage of the value for the untreated control wells. The values presented are the average of two replicates and are representative of two independent experiments. Download FIG S2, TIF file, 0.7 MB. Copyright © 2020 DeJarnette et al. 2020 DeJarnette et al. This content is distributed under the terms of the Creative Commons Attribution 4.0 International license.

**FIG 8:**
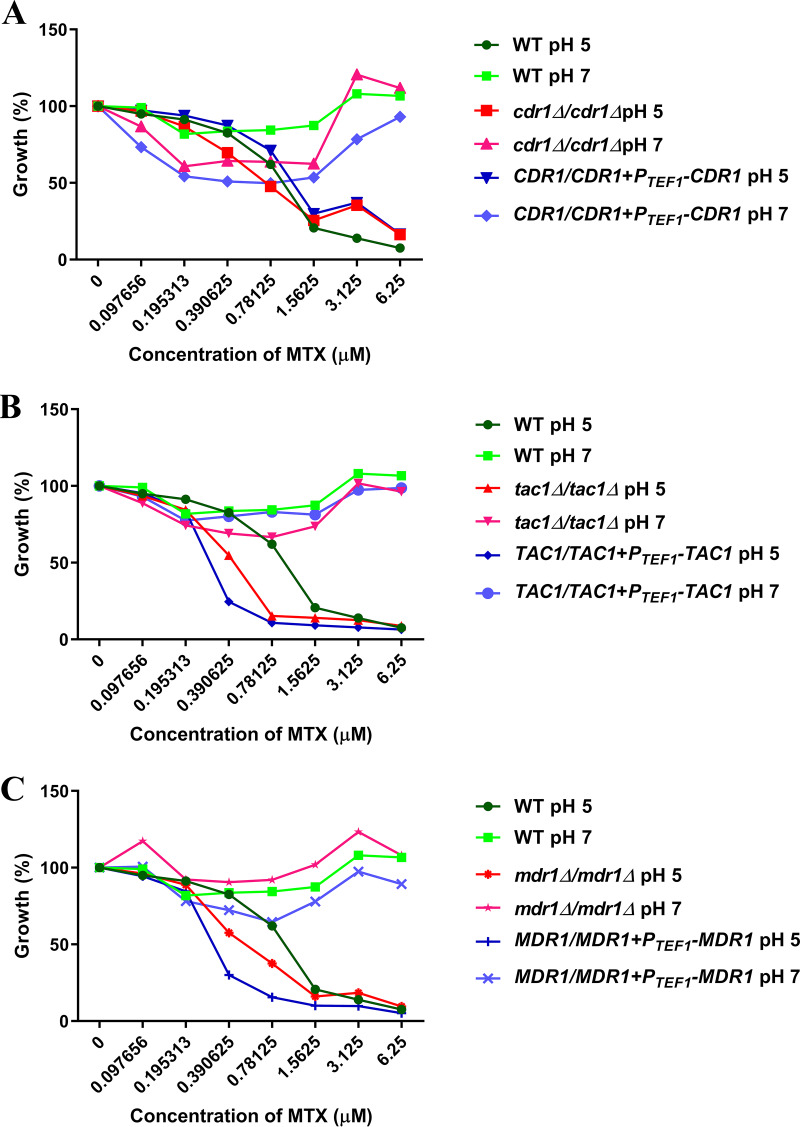
Efflux pump expression does not substantially alter Candida albicans susceptibility to methotrexate. C. albicans *cdr1*Δ/Δ (A), *tac1*Δ/Δ (B), or *mdr1*Δ/Δ (C) strains, or that overexpress *CDR1* (A), *TAC1* (B), or *MDR1* (C) from the *P_TEF1_* promoter, were subcultured to approximately 1 × 10^4^ cells/ml into YNB medium at pH 5 or pH 7 supplemented with a range of MTX concentrations. After 48 h at 30°C, growth was quantified as the OD_600_ value and expressed as a percentage of the value for the untreated wells. The values presented here are the average of two replicates and are representative of two independent experiments. WT, wild type.

We therefore examined if the antifungal activity of MTX was affected by the most important drug efflux mechanisms in C. albicans. The susceptibility of a C. albicans *cdr1Δ*/*Δ* mutant, lacking an ATP-dependent ABC family transporter, as well as that of a *tac1Δ*/*Δ* mutant, lacking a zinc cluster transcription factor that activates the expression of the Cdr1p and Cdr2p drug efflux pumps (42), is not significantly different from isogenic or wild-type control strains in YNB at pH 5 (MIC_50_ of 0.39 μM) or pH 7 (MIC_50_ > 6.25 μM) (Fig. 8A and B). Similarly, we found that the susceptibility of an *mdr1Δ*/*Δ* mutant, lacking a major facilitator superfamily transporter, which is driven by the proton motive force at the plasma membrane and which has been previously implicated in methotrexate efflux (43, 44), was not significantly different from *MDR1^+^* control strains in YNB medium at pH 5 or 7 (Fig. 8C). The susceptibility of strains engineered to overexpress either the Cdr1p or Mdr1p efflux pumps (42) was also unaffected. This lends further support for the hypothesis that the pH-dependent antifungal activity of MTX is a result of differential cellular uptake or permeability.

**FIG 9:**
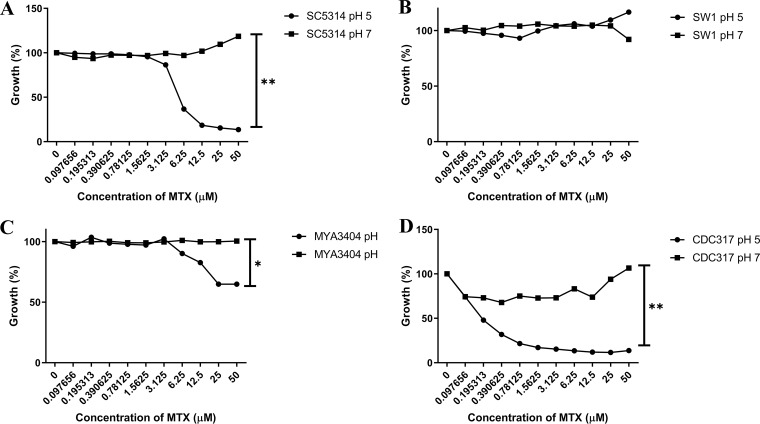
The pH-dependent antifungal activity of methotrexate is species specific. Strains of C. albicans (A), C. glabrata (B), C. tropicalis (C), and C. parapsilosis (D) were seeded into YNB medium buffered at pH 5 or pH 7 at approximately 1 × 10^4^ cells/ml in the presence of a range of MTX concentrations. After 48 h at 30°C, growth was quantified as the OD_600_ and expressed as a percentage of the value for the untreated controls. The values presented here are the average of two replicates and are representative of two experiments. The susceptibility to MTX at the highest concentration at each pH was compared to that of the other pH values using a two-tailed Student's *t* test (*, *P* < 0.01; **, *P* < 0.0001).

To determine if MTX’s pH-dependent activity was specific to C. albicans, we tested its antifungal activity upon other fungal species in YNB medium at pH 5 and pH 7. As in C. albicans, MTX showed modest and pH-dependent growth inhibition on Candida tropicalis and Candida parapsilosis. In contrast, Candida glabrata was insensitive at either pH (Fig. 9).

### Established DHFR inhibitors are unable to inhibit Dfr1p within Candida albicans cells.

Finally, we tested a panel of additional antifolates to examine the relationship between their antifungal potencies and capacities to inhibit C. albicans DHFR. The enzyme was purified using a 6×His tag (45, 46), and activity was detected by measuring the conversion of NADPH to NADP^+^ at an optical density at 340 nm (OD_340_) (34). Dose-response experiments confirmed MTX to be a potent inhibitor of C. albicans DHFR, while trimethoprim (TMP) had no activity (Table 1), as previously reported (47). While proguanil (PRO) also lacked activity (50% inhibitory concentration [IC_50_] > 10 μM), both pyrimethamine (PYR) and pemetrexed (PTX) inhibited C. albicans DHFR activity. Consistent with the biochemical data, neither TMP nor PRO was able to inhibit C. albicans growth in YNB medium buffered to either pH 5 or 7 (MIC >25 μM). PYR also failed to inhibit C. albicans growth at either pH (Fig. S3), suggesting that the compound is unable to access DHFR in whole cells. Similar to MTX, PTX possessed antifungal activity at pH 5 but not at pH 7 in YNB medium, presumably reflecting pH-dependent cell permeability.

**TABLE 1:** Antifolates inhibit the activity of Candida albicans dihydrofolate reductase

Drug	IC_50_ (μM)
Methotrexate	0.001
Trimethoprim	>10
Pyrimethamine	0.1
Pemetrexed	1.0
Proguanil	>10

10.1128/mSphere.00374-20.3 FIG S3 Methotrexate and pemetrexed have pH-dependent antifungal activity upon Candida albicans. C. albicans strain SC5314 was subcultured to ∼1 × 10^4^ cells/ml in YNB medium at pH 3, 4, 5, 6, or 7, with increasing concentrations of MTX (A), sulfamethoxazole (SMX) (B), pyrimethamine (PYR) (C), pemetrexed (PTX) (D), proguanil (PRO) (E), or trimethoprim (F). After 48 h, growth was quantified as the OD_600_ and expressed as a percentage of the value for the DMSO-treated control. The results presented here are the averages of two replicates and are representative of two independent experiments. Download FIG S3, TIF file, 2.7 MB. Copyright © 2020 DeJarnette et al. 2020 DeJarnette et al. This content is distributed under the terms of the Creative Commons Attribution 4.0 International license.

10.1128/mSphere.00374-20.4 FIG S4 Schematic representation of the folate biosynthetic pathway. Download FIG S4, TIF file, 0.2 MB. Copyright © 2020 DeJarnette et al. 2020 DeJarnette et al. This content is distributed under the terms of the Creative Commons Attribution 4.0 International license.

## DISCUSSION

While folate biosynthesis has been successfully targeted to develop antibacterial, antiprotozoal, and antineoplastic therapies, there have been relatively few efforts to develop antifungals that target this pathway. Although a combination of trimethoprim and sulfamethoxazole is used clinically to treat *Pneumocystis* pneumonia and *Paracoccidioides* infections (27, 48), many of the most important fungal pathogens are largely insensitive to conventional antifolate drugs (28, 49, 50). A handful of studies have attempted to produce derivatives of conventional antifolates with enhanced antifungal potency but have failed to yield derivatives with the requisite properties of a viable antifungal drug. For example, an entire series of sulfone compounds completely lack antifungal activity against whole C. albicans cells, despite potent inhibition of fungal DHPS *in vitro* (49). Similarly, a large series of diaminopyrimidines, which inhibit bacterial and human DHFR, have little activity against whole C. albicans cells despite potent inhibition of fungal DHFR in cell-free assays (49). DHFR inhibitors based on either quinazoline (51) or pteridine (52) ring scaffolds have potent but pH-dependent antifungal activity *in vitro*, which likely explains their lack of efficacy in an animal model of infection (52). One factor complicating the interpretation of these findings is that these studies often used different culture conditions or media. We therefore considered that the lack of success could be accounted for by infectious species, such as C. albicans, acquiring exogenous sources of folate from certain growth media or under specific conditions *in vitro* and/or from mammalian tissue *in vivo*. This would bypass the need for *de novo* folate biosynthesis and therefore render antifolate drugs ineffective as antifungals. However, our results using the doxycycline-repressible *DFR1* strain revealed that DHFR and, by inference, *de novo* production of THF are essential for C. albicans growth in a variety of culture conditions, including within blood serum as well as in the mammalian host. Aside from extremely high concentrations of folinic acid, medium supplements of various forms of folate, including the DHFR product THF, were not able to restore growth upon loss of DHFR expression. Thus, DHFR is a valid and potentially efficacious target for antifungal development.

These conclusions are further supported by the fact that methotrexate has relatively potent antifungal activity against C. albicans in the lower-pH range. In contrast, doxycycline-mediated suppression of DHFR activity in the *P_TET98_-DFR1* strain occurred less rapidly, requiring multiple passages to arrest fungal growth completely. This is likely a consequence of an indirect mechanism, acting through suppression of *DFR1* transcription, rather than direct inhibition of the fungal enzyme’s activity. Nonetheless, the symptoms observed within mice infected with the *P_TET98_-DFR1* strain resolved fairly quickly upon doxycycline treatment, indicating that the fungus is highly sensitive to perturbation of THF production *in vivo*. We conclude that an antifungal drug acting through inhibition of DHFR has the potential to provide a highly efficacious antifungal therapy against C. albicans. However, given that Dfr1p is present in humans and fairly well conserved between mammals and fungi, selectivity is likely to be an issue. Interestingly, Anderson and colleagues (18, 53) described trimethoprim derivatives with an extended central linker with dramatically enhanced potency against C. glabrata DHFR. However, despite exhibiting selective inhibition of the fungal enzyme over the human enzyme in cell-free biochemical assays, toxicity to mammalian cells remained an issue (54). Accordingly, assuming that inhibition of earlier steps of the folate (FOL) biosynthetic pathway results in similar antifungal efficacy, it may make more sense to target the enzymes absent from the mammalian host.

Folate synthesis starts with the conversion of GTP to dihydroneopterin triphosphate by GTP cyclohydrolase I (Fol2p), which is subsequently condensed with *para*-aminobenzoic acid (PABA) to form dihydropteroate by the sequential action of dihydroneopterin aldolase (DHNA), hydroxymethyldihydropterin pyrophosphokinase (HPPK), and dihydropteroate synthase (DHPS) (55). In yeast, the last three enzyme activities are provided by a single trifunctional protein, Fol1p, while in filamentous fungi HPPK and DHPS form a bifunctional enzyme (38). Dihydrofolate synthase (DHFS; Fol3p) adds a single l-glutamate residue to produce DHF, which is subsequently reduced by DHFR (Dfr1p) to produce THF that can accept 1C substituents. Notably, mammals completely lack four of the core enzymes required to produce folate, DHNA, HPPK, DHPS (Fol1p), and DHFS (Fol3p). In the noninfectious yeast Saccharomyces cerevisiae, the *FOL1* and *FOL3* genes are both essential for viability under normal culture conditions (38, 56). A recent large-scale study using transposon mutagenesis also predicted *FOL1* to be essential in C. albicans (57), and an unpublished study has indicated that the bifunctional *FOL1* ortholog is essential in Aspergillus fumigatus (58). Nonetheless, these enzymes are surprisingly poorly studied in infectious fungi or, indeed, in S. cerevisiae.

The fact that the doxycycline-repressible *DFR1* strain is unable to grow in any growth medium tested (including those supplemented with THF) under repressing conditions is consistent with conventional antifolates lacking activity upon the target enzyme in whole cells rather than with an inherent capacity of C. albicans to bypass the need for folate biosynthesis. Given that both sulfa-based DHPS inhibitors and various DHFR inhibitors have demonstrated relatively potent inhibition of the respective fungal enzymes in cell-free assays (34, 47, 49, 52, 53), the most likely challenge in deriving antifungals that target folate biosynthesis is achieving sufficient intracellular accumulation. In the case of mammalian cells, DHFR inhibitors such as MTX, which are structurally related to folic acid itself, enter primarily through the endogenous folate transport system (59). While the lack of a known folate transport system, in theory, renders fungi vulnerable to antifolates, it simultaneously raises challenges in promoting the uptake of folate analogs. We consider two possible explanations for the pH-dependent antifungal activity of MTX. First, fungi could potentially encode a pH-dependent folate transport system that enables MTX uptake in more acidic conditions. However, we do not favor this explanation as such a system would be expected to facilitate the uptake of folates in the medium itself simultaneously and thus bypass the need for *de novo* synthesis. Second, and perhaps more likely, MTX uptake in fungi could occur solely through passive diffusion across the plasma membrane, with the pH dependence simply reflecting the ionization state of MTX. The pKa of MTX is 4.7 (60); thus, deprotonation of the associated carboxylic acid group of MTX at higher pH would confer a negative charge that may render it membrane impermeable. Either way, we found that the best-characterized drug efflux mechanisms in C. albicans, the proton-driven Mdr1p and ATP-driven Cdr1p and Cdr2p transporters, had little impact on the sensitivity of strain SC5314 to MTX and therefore do not explain the lack of antifungal activity at neutral pH. Thus, existing drugs that inhibit DHFR cannot provide effective antifungals for two main reasons. First, the fungal enzyme is insensitive to these drugs, presumably due to evolutionary divergence from the DHFR enzyme of their intended target species. For example, while trimethoprim apparently possesses some antifungal activity against Pneumocystis jirovecii, given its clinical efficacy, C. albicans DHFR is insensitive (47). Second, many conventional DHFR inhibitors are unable to access the enzyme within the intact fungal cell, presumably due to limiting membrane permeability. This likely accounts for the lack of antifungal activity of pyrimethamine and the pH-dependent activity of MTX and pemetrexed.

C. glabrata was insensitive to MTX at any pH, which indicates that either the Dfr1p enzyme is structurally distinct or that this species has a different acquisition or permeability of folates. Previous studies have shown that the structure of Dfr1p is conserved between C. glabrata and C. albicans (CgDfr1p and CaDfr1p, respectively) (61). However, this same study established that while an inhibitor could inhibit both CgDfr1p and CaDfr1p, this enzyme inhibition did not always correlate with fungal growth inhibition, nor did the inhibition correlate between species (62). Therefore, we conclude that the differential in MTX pH-dependent activity may be a result of differential uptake of antifolates.

In summary, our results demonstrate that the *de novo* synthesis of THF is absolutely required for C. albicans to cause disease within the mammalian host and that DHFR is a valid and potentially efficacious target for antifungal development. However, given the structural similarity of fungal and mammalian DHFR and the difficulties in producing DHFR inhibitors that are active upon whole fungal cells, we propose that efforts to exploit the FOL pathway for antifungal development should focus upon the biosynthetic enzymes that have not yet been the subject of significant investigation and which are completely absent from mammals. In addition, this may enhance the chances of discovering novel antifolate scaffolds that have activity upon whole fungal cells.

## MATERIALS AND METHODS

### Growth conditions.

C. albicans was routinely grown on yeast extract-peptone-dextrose (YPD) agar plates at 30°C, supplemented with 50 μg/ml uridine for *ura3Δ*/*Δ* strains. Selection of C. albicans transformants was carried out on minimal YNB medium (6.75 g/liter yeast nitrogen base without amino acids, 2% dextrose, 2% Bacto agar) supplemented with the appropriate auxotrophic requirements described for S. cerevisiae (63) or with 50 μg/ml uridine.

### Candida albicans strain construction.

BWP17 (64) was kindly provided by Aaron Mitchell (Carnegie Mellon University). C. albicans was transformed with DNA constructs using a lithium acetate procedure (65). The parental strain, BACTR31, was constructed by transforming BWP17 with pDUP3 (66) that contains the tetracycline repressor protein (TetR) from Nakayama and colleagues (33). To generate this plasmid, *tetR* from THE1 (33) was PCR amplified using primers TetRHap4ADF-SalI and TetRHap4ADR-MluI and then cloned into pDUP3 between SalI and MluI sites. This pDUP3::*tetR* plasmid was linearized using NaeI and transformed into BWP17.

The *DFR1* gene deletion cassette was amplified using the primer set DFR1DISF and DFR1DISR and plasmid pRSARG4ΔSpeI (*ARG4* selection marker) as a template and transformed into BACTR31. Correct integration of the gene deletion cassette to replace one *DFR1* allele was confirmed by diagnostic PCR, using primers ARG4INTR2 and DFR1AMPR-SacI as well as ARG4INTF2 and DFR1AMPF-KpnI. To make the *P_TETO_-DFR1* strain, we generated promoter replacement cassettes by PCR amplifying the tetracycline-repressible promoter from the plasmids p97CAU, p98CAU, or p99CAU (33) using the primers DFR1TETF and DFR1TETR. Each cassette was transformed into the BACTR31-derived *DFR1*/*dfr1Δ*::*ARG4* heterozygote, and the presence of *P_TETO_* was determined by diagnostic PCR using TETOSEQF and DFR1DETR. The absence of a wild-type allele was determined by diagnostic PCR using DFR1AMPF-KpnI and DFR1DETR. The strains were then made prototrophic by transforming pGEMHIS1 (32) linearized with NruI.

### RNA isolation and qRT-PCR.

RNA was isolated using the hot-phenol method of RNA isolation described previously (35). This RNA pellet was washed with 500 μl of 70% ice-cold ethanol and collected by centrifugation. The RNA pellet was resuspended in DNase/RNase-free H_2_O. cDNA was synthesized from total RNA using a Verso cDNA synthesis kit (Thermo Scientific) in accordance with the manufacturer’s instructions. Synthesized cDNA was used for both the amplification of *ACT1* and the gene of interest by PCR, using SYBR green PCR master mix, according to the manufacturer’s instructions. Gene-specific primers were designed using the PrimerQuest tool from Integrated DNA Technologies (IDT) and synthesized by IDT and are listed in Table S1 in the supplemental material. The PCR conditions consisted of an initial denaturation at 95°C for 10 min, followed by 40 cycles of denaturation (95°C for 15 s), annealing (60°C for 30 s), and extension (72°C for 37 s). Software (version 1.2.3) for the 7500 Sequence Detection System from Applied Biosystems was used to determine the dissociation curve and threshold cycle (*C_T_*). The 2^−ΔΔ^*^CT^* method was used to calculate changes in gene expression levels among the strains (67). All experiments included both biological and technical replicates in triplicate.

10.1128/mSphere.00374-20.5 TABLE S1 Oligonucleotides used in this study. Engineered restriction enzyme sites are underlined. Download Table S1, DOCX file, 0.01 MB. Copyright © 2020 DeJarnette et al. 2020 DeJarnette et al. This content is distributed under the terms of the Creative Commons Attribution 4.0 International license.

### Antifungal susceptibility testing.

Stock solutions of MTX were prepared at 10 mM in dimethyl sulfoxide (DMSO) and diluted as needed in the same solvent. Each C. albicans strain was grown overnight in YPD medium at 30°C and diluted to approximately 1 × 10^4^ cells/ml in YNB medium or RPMI 1640 medium, and 100 μl of each cell suspension was transferred to the wells of a round-bottom 96-well plate. An additional 100 μl of YNB or RPMI 1640 medium containing 2× the final desired concentration of MTX was then added to each well. The final concentration of DMSO was 0.5% for all treatments. Plates were then incubated at 30°C before growth was quantified after 24 and 48 h by measuring the OD_600_ using a Cytation 5 plate reader (Bio-Tek Instruments, Inc.). MIC_50_ values were identified visually using the CLSI antifungal susceptibility assay and then confirmed by quantifying growth by the OD_600_ value. The growth of each strain at each drug concentration was then expressed relative to that of the DMSO-alone control. The susceptibility to MTX at the highest concentration at each pH was compared to that of the other pH values using a two-tailed Student's *t* test.

### Mouse model of disseminated candidiasis.

All animal experiments were done according to protocols approved by the University of Tennessee Institutional Animal Care and Use Committee. Groups of 6- to 8-week-old female BALB/c mice (Charles River Laboratories) were randomly assigned to one of three treatment groups 3 days before infection. Group 1 (minus doxycycline) was provided 20 g of DietGel (clear H_2_O) per day, per cage of 4 mice, while group 2 (plus doxycycline) was provided the same diet supplemented with 2 mg/ml of doxycycline hyclate. Group 3 (posttreatment) mice were given the DietGel without doxycycline until 24 h after infection and were administered 10 mg/kg of doxycycline through oral gavage at 12, 24, and 36 h postinfection. Treatment was maintained daily for the duration of the experiment (up to 12 days postinfection). Each C. albicans strain was grown overnight in YPD cultures at 30°C (200 rpm). Cells were washed twice in sterile endotoxin-free phosphate-buffered saline (PBS), and cell density was determined using a hemocytometer. Each strain was then diluted to 5 × 10^6^ cells/ml in sterile PBS, and 0.1 ml of each cell suspension was inoculated into the lateral tail vein of mice from each treatment group. Viable cell counts of each inoculum were confirmed by plating appropriate dilutions onto YPD agar plates and counting the number of colonies formed after 48 h. Mice were monitored for 12 days, and those showing distress were euthanized. Survival data were plotted on a Kaplan-Meier curve and analyzed by a log rank (Mantel-Cox) test using GraphPad Prism, version 8.00. The kidneys from each mouse were extracted, weighed, and homogenized in PBS. Serial dilutions of kidney homogenate were plated on YPD agar plates containing 50 μg/ml of chloramphenicol. The number of CFU/gram of kidney tissue was determined from the number of colonies on the plates after 48 h.

### Purification of the Candida albicans dihydrofolate reductase.

The C. albicans *DFR1* open reading frame (ORF) sequence was retrieved from the *Candida* Genome Database (68) and was codon optimized for expression in Escherichia coli. The optimized *DFR1* ORF was then cloned into pET15b (69) to allow propagation and expression of C. albicans Dfr1p in E. coli. Cultures of E. coli transformed with pET15b-CaDfr1p (Biomatik) were grown overnight in LB plus ampicillin. The culture was diluted 1:100 into fresh medium and incubated at 35°C for 6 h; then Dfr1p expression was induced with isopropyl-β-d-thiogalactopyranoside (IPTG) at a final concentration of 0.5 mM for 16 h. Cells were collected by centrifugation and resuspended in binding buffer (6 g/liter Tris base, pH 7.5, 8.8 g/liter NaCl, 1 mM dithiothreitol [DTT], 0.68 g/liter imidazole, 5% glycerol, 1× protease inhibitor). Resuspended cells were lysed with 1 mg/ml chicken white lysozyme (Sigma) for 60 min at 4°C. Then, Triton X-100 and NaCl were added to 0.1% and 0.5 M, respectively. The lysate was then centrifuged, and the supernatant was collected. The supernatant was added to nickel resin beads (Sigma) and incubated at 4°C for 30 min with rotation. The buffer was removed, and the beads were washed twice with binding buffer. Then the beads were suspended in elution buffer (6 g/liter Tris base, pH 7.5, 8.8 g/liter NaCl, 1 mM DTT, 34 g/liter imidazole, 5% glycerol) and incubated at 4°C for 30 min. The elution was removed and then run on SDS-PAGE gels (Sigma) to confirm the presence of CaDfr1p. SDS-PAGE gels were run according to the manufacturer’s protocol.

### Dfr1p enzyme assay.

The enzymatic activity of C. albicans Dfr1p was determined using a dihydrofolate reductase assay (Sigma). The manufacturer’s protocol was adapted to the fungal enzyme by scaling down the volume to 100-μl reaction mixtures with 88 μl of buffer, 6 μl of a 1 mM NADPH stock, 5 μl of a 1 mM DHF stock, and 1 μl of 3-mg/ml purified enzyme. The loss of NADPH was quantified as the decrease in absorption at the OD_340_ over a 30-min time frame. The IC_50_ was calculated by converting the change in the OD_340_ value per minute into the amount of THF produced per minute and then compared to the level in an untreated control.
