# Supplementary figures and images for: Dihydrofolate Reductase Is a Valid Target for Antifungal Development in the Human Pathogen Candida albicans
Source: mSphere. 2020 Jun 24;5(3):e00374-20. doi: 10.1128/mSphere.00374-20 (PMC7316490; doi:10.1128/mSphere.00374-20)

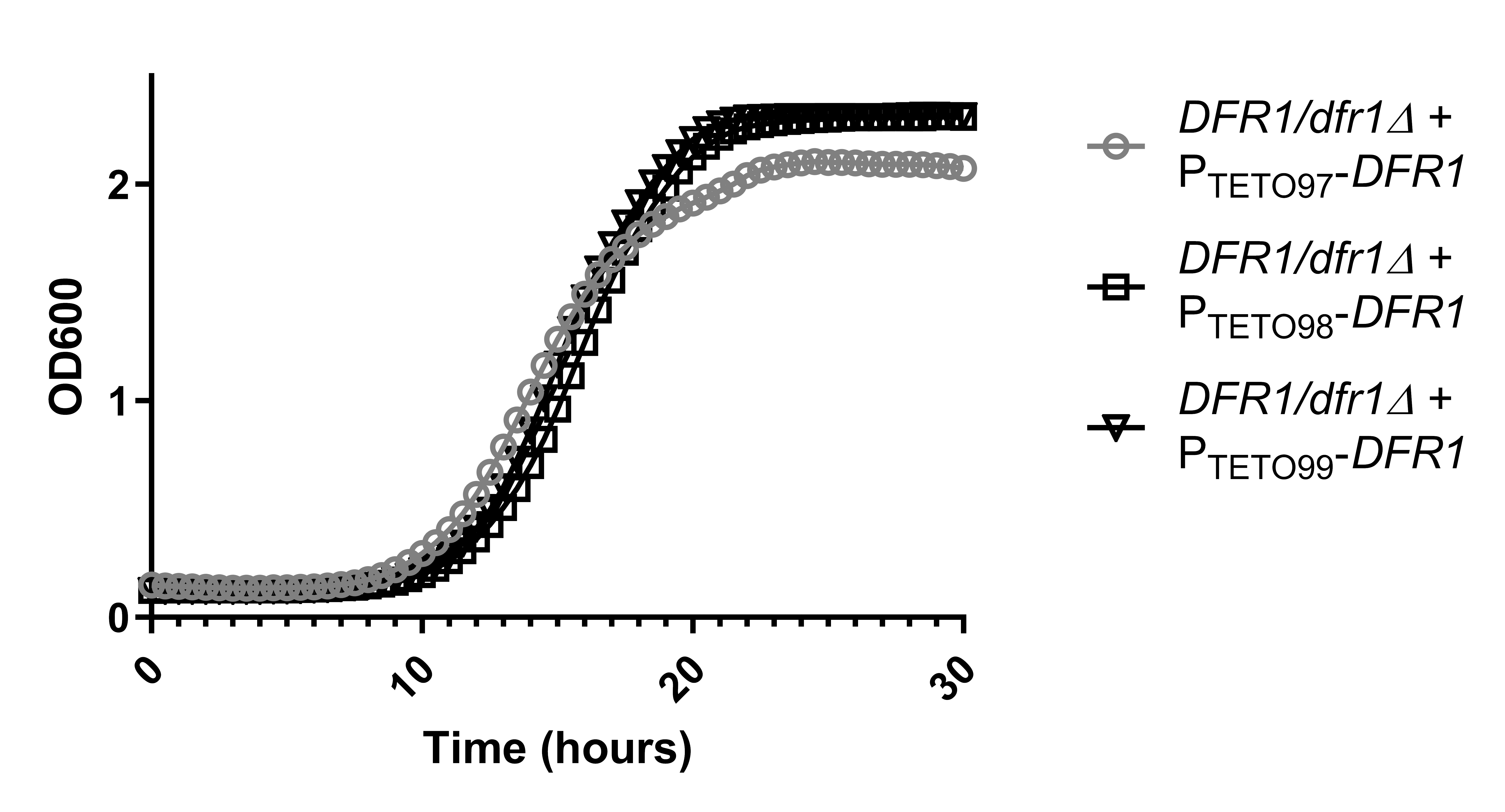

Supplement: FIG S1 [file mSphere.00374-20-sf001.tif]

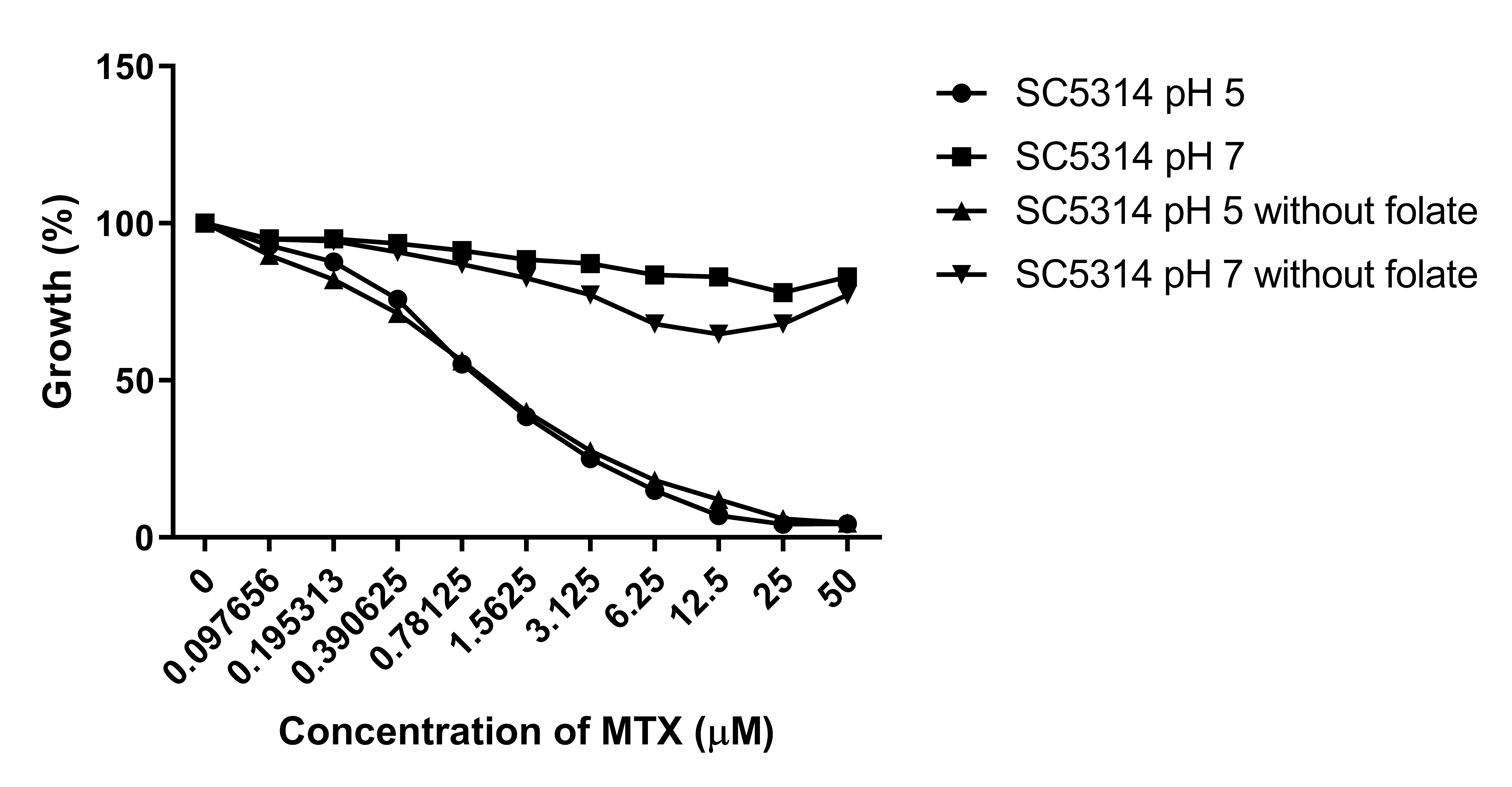

Supplement: FIG S2 [file mSphere.00374-20-sf002.tif]

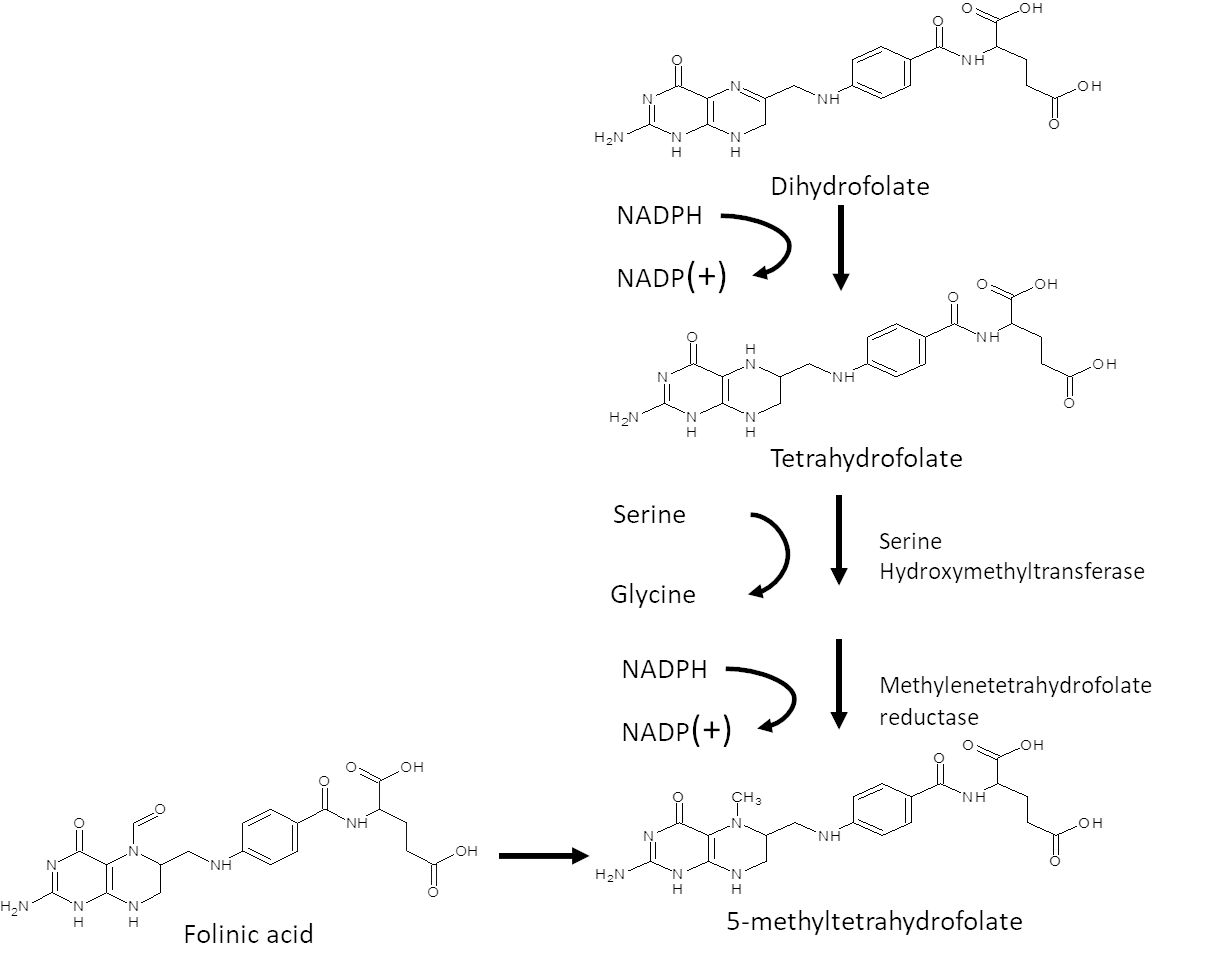

Supplement: FIG S4 [file mSphere.00374-20-sf004.tif]
