# Supplementary material for: Dihydrofolate Reductase Is a Valid Target for Antifungal Development in the Human Pathogen Candida albicans
Source: mSphere. 2020 Jun 24;5(3):e00374-20. doi: 10.1128/mSphere.00374-20 (PMC7316490; doi:10.1128/mSphere.00374-20)
Supplement: TABLE S1 [file mSphere.00374-20-st001.docx]

| Primer | **Sequence 5’→3’*** |
| --- | --- |
| DFR1DISF | TTCTTGTTTGATTGAAAAAAAACTTTCACCACTAATCTAGAACTTCACGAATAGACAACAGTTTTCCCAGTCACGACGTT |
| DFR1DISR | TTATTTTCTTGTCCATAGCGTATAATTATAGGTAAAATCACCTTCCTTGATATCGTCTTTGTGGAATTGTGAGCGGATA |
| DFR1TETOF | TGGACTTAGGGGTACTTGGCCAGGTATTGTATTTTTATCAGGTTATTCATTGTTGCACTCTAGGGTAATACGACTCACTATAGGG |
| DFR1TETOR | CTTTGTATCCAATTCCCAAAGCAGGCTTTAATGCCGCGACAATGATTGCAACATTTGGTTTCAGCATCTAGTTTTCTGAGATAAAGCTG |
| ARG4INTF2 | AAGCTAGTGTGGAAAGAAGAG |
| ARG4INTR2 | AATGACTGAATTATGTCGGTC |
| DFR1AMPF-KpnI | TCAGGTACCTTGAGTTGTGGACTTAGTGGG |
| DFR1AMPR-SacI | TCAGAGCTCCGACTTTGGAGAGAGAACAGC |
| TETODETF | GTTGACACTTGTAAATAAGCG |
| DFR1DETR | TTGGTCCAACTTTCCAATGGG |
| TetRHap4ADF-Sal1 | TCAGTCGACATGTCTAGATTAGATAAAAGTAAAG |
| TetRHap4ADR- Mlu1 | TCAACGCGTCAACTCAATTGAGGTTACTCGG |
| DFR1q1 | TGTTGCAATCATTGTCGCGG |
| DFR1q2 | CCTTACGGAGTCTCCAAGGC |
| ACT1q1 | TTGGATTCTGGTGATGGTGTTA |
| ACT1q2 | TCAAGTCTCTACCAGCCAAATC |
